# A simplified fractional order impedance model and parameter identification method for lithium-ion batteries

**DOI:** 10.1371/journal.pone.0172424

**Published:** 2017-02-17

**Authors:** Qingxia Yang, Jun Xu, Binggang Cao, Xiuqing Li

**Affiliations:** 1 State Key Laboratory for Manufacturing Systems Engineering, School of Mechanical Engineering, Xi'an Jiaotong University, Xi'an, Shaanxi, China; 2 Engineering Research Center of Tribology and Material Protection of Ministry of Education, Henan University of Science and Technology, Luoyang, Henan, China; Beihang University, CHINA

## Abstract

Identification of internal parameters of lithium-ion batteries is a useful tool to evaluate battery performance, and requires an effective model and algorithm. Based on the least square genetic algorithm, a simplified fractional order impedance model for lithium-ion batteries and the corresponding parameter identification method were developed. The simplified model was derived from the analysis of the electrochemical impedance spectroscopy data and the transient response of lithium-ion batteries with different states of charge. In order to identify the parameters of the model, an equivalent tracking system was established, and the method of least square genetic algorithm was applied using the time-domain test data. Experiments and computer simulations were carried out to verify the effectiveness and accuracy of the proposed model and parameter identification method. Compared with a second-order resistance-capacitance (2-RC) model and recursive least squares method, small tracing voltage fluctuations were observed. The maximum battery voltage tracing error for the proposed model and parameter identification method is within 0.5%; this demonstrates the good performance of the model and the efficiency of the least square genetic algorithm to estimate the internal parameters of lithium-ion batteries.

## Introduction

In recent years, with the rapid development of electric vehicle (EV) technology, lithium-ion batteries have been attracting much attention because of their superior performance [1]. Unfortunately, unexpected system failures usually occur due to environmental impacts, dynamic loading, and especially battery degradation [2]. Some special methods have been developed to study the failure of lithium-ion batteries (LIBs), including the short circuit test method [3], the internal parameter monitoring method [4], and so on. Xu et al. [5] investigated the electrochemical failure behaviors of lithium-ion batteries with different states of charge (SOC) underpinned by the short circuit phenomenon, and proposed a nominal stress–strain curve to further quantify the short circuit occurrence with mechanical behavior. Yet, the short circuit test method was destructive for the power system in EV. The internal parameters of lithium-ion batteries can reflect the main characteristics of batteries in different states [6], thus, constant monitoring of these parameters could be useful to evaluate the battery performance. However, the electrochemical process of lithium-ion batteries is so complex that the internal parameters cannot be measured directly, so an accurate model and a highly precise parameter identification algorithm are required [7].

In recent years attempts have been made to build models to estimate the internal parameters of lithium-ion batteries, such as electrochemical models [8,9], mechanical models [10,11] and equivalent circuit models (ECMs) [12,13]. The electrochemical models are usually used to describe battery electrochemical properties combined with the mechanical models. For example, Liu et al. [11] proposed a coupling electrochemical-circuit model to predict battery penetration process, and designed a series of penetration test to validate the computational model. ECMs consist of a series of electronic components including resistors, capacitors, and inductors. First-order resistance-capacitance (1-RC) [3,14] and second-order resistance-capacitance (2-RC) models [15,16] are the most commonly used ECMs; yet, high-order RC models have been reported to be much more accurate. For example, a relaxation model has been proposed by Schmidt et al. [17], in which tens or hundreds of parallel RC circuits were employed to represent the distributed relaxation times. Besides, electrochemical models such as pseudo-two-dimensional models [18], single particle models, and extended single particle models [19] are more accurate than ECMs; however, they require a large number of parameters that cannot be measured.

Fractional order models (FOMs) [20,21], derived from the above-mentioned models, have recently attracted increasing interest in this field. Wang et al. [22] presented a FOM for lithium-ion batteries that showed higher accuracy for voltage tracing under different conditions compared with the commonly used 1-RC models. Moreover, Xu et al. [20] reported a FOM in which a fractional order calculus (FOC) was used to describe the constant phase element (CPE) and Warburg element, and the differentiation order of the Warburg element was fixed at 0.5. The models mentioned above have been widely used, but they do not provide satisfactory estimation results. Hence, it is still a challenge to achieve a battery model with high accuracy and computational efficiency.

In addition, parameter identification methods, required for the characterization of lithium-ion batteries, have been widely investigated [23–25]. Joel et al. [26] proposed a parameter identification method based on a genetic algorithm (GA) for a LiFePO_4_ cell electrochemical model. Cell voltage and power were estimated with a relative error of 5%, a value higher than expected. Moreover, Chen et al. [27] described a GA-based parameter identification method for a 2-RC model with a sufficiently precise margin of error; however, the application of a GA-based identification method to a fractional order impedance model (FIM) has not yet been reported.

In this paper, a simplified FIM for lithium-ion batteries and the corresponding parameter identification method are presented. The simplified FIM is derived from the analysis of electrochemical impedance spectroscopy (EIS) and hybrid pulse power characteristic (HPPC) test data, and the model parameters are identified using an equivalent tracking system through a least square genetic algorithm (LSGA). The effectiveness and accuracy of the proposed model and the corresponding parameter identification method are verified by experiments and simulations.

## Fractional impedance model

### EIS and ECM of lithium-ion batteries

EIS is one of the best methods to describe the dynamic characteristics of batteries [28]. In the EIS test, the sinusoidal AC signals of different frequencies and amplitudes were applied to electrochemical systems, and the signal feedback in the frequency domain was obtained. The EIS measurements provided accurate impedance values at different frequencies, and it is convenient to determine the battery dynamic response via the EIS test. Therefore, the EIS test could be used to describe the properties of battery system.

**Fig 1:**
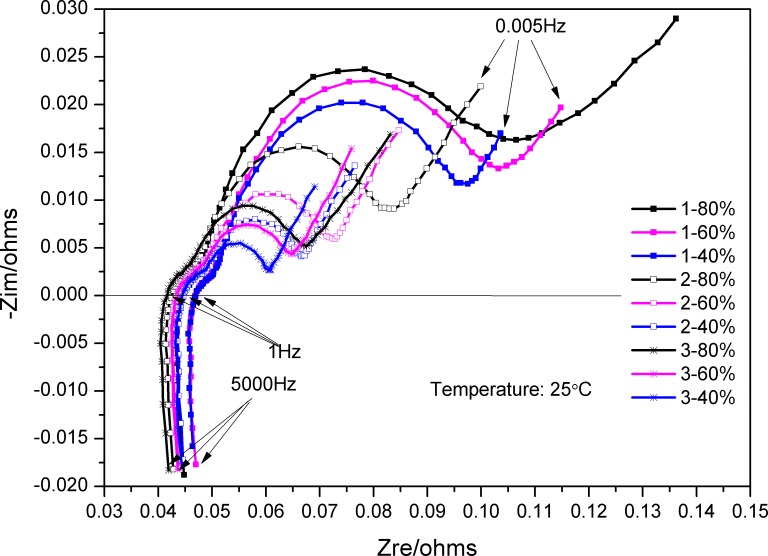
EIS curves of lithium-ion batteries with different SOC and maximum discharge capacities.

In this study, commercially available Panasonic NCR18650 lithium-ion batteries with 2.9 Ah capacity, nickel manganese cobalt oxide cathode and graphite anode, designed for electric vehicle applications, were used. The specifications of the lithium-ion batteries are shown in Table 1A in S1 File. The EIS of three batteries were measured using a Princeton electrochemical workstation at room temperature 25°C, and the batteries were test under different maximum discharge capacities and SOC conditions as shown in Table 1. Results of the EIS test are presented in Fig 1.

**Table 1:** EIS test conditions.

Battery number	Capacity/mAh	SOC		
001	2422	80%	60%	40%
002	2661	80%	60%	40%
003	2855	80%	60%	40%

**Fig 2:**
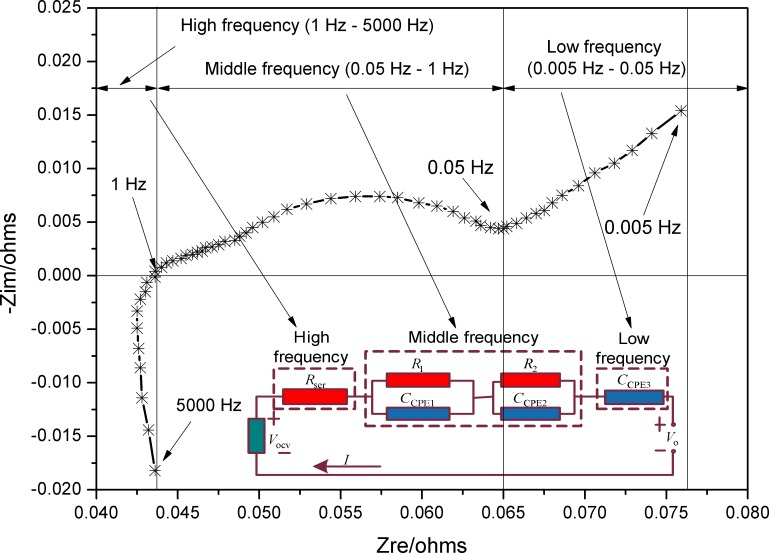
ECM based on the EIS response of the lithium-ion battery with 2855 mAh capacity and 60% SOC.

It can be seen from Fig 1 that the EIS curves are similar in shape, but the Zre and–Zim values change with the test condition at the same frequency. The EIS of the battery with a capacity of 2855 mAh and 60% SOC (Fig 2), recorded in the frequency range 5 mHz–5 KHz, consists of three sections, namely, a high-frequency, a mid-frequency, and a low-frequency section.

In the high-frequency region (1 Hz–5000 Hz), the plot consists of a vertical straight line, associated with an element, followed by a depressed semicircle, indicating a resistor parallel to a CPE. In the mid-frequency region (0.05 Hz–1 Hz), the impedance spectrum shows a depressed semicircle, which could be modeled by a parallel resistor/CPE combination; the parallel combination could represent the charge-transfer reaction on the solid electrolyte interphase layer described by the Butler-Volmer equation. In the low-frequency region (0.005 Hz–0.05 Hz), the EIS curve looks like to be a straight line with a constant slope of 1, which could be expressed as a Warburg element, previously modeled by a CPE element [20]. The EIS response in the low-frequency section could be used to reflect the diffusion dynamics inside spherical particles determined by Fick's second law. The impedance spectrum was normalized to obtain an ECM, as shown in Fig 2.

**Fig 3:**
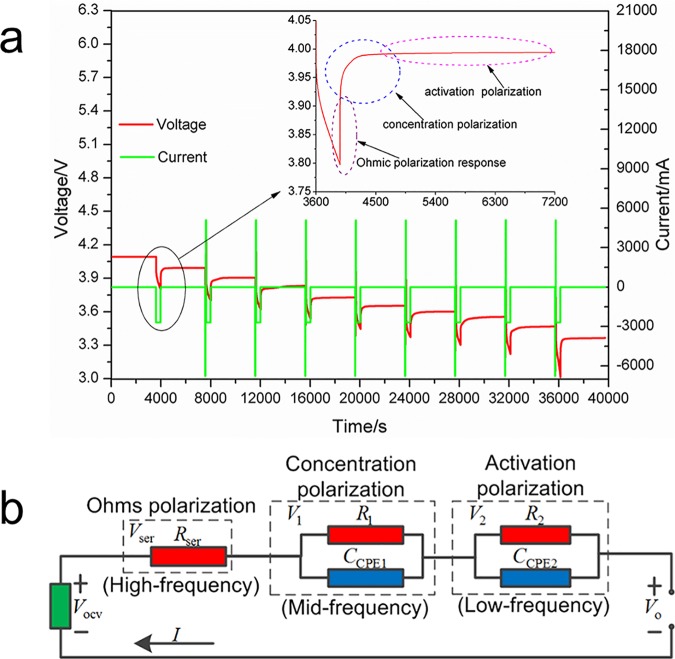
(a) HPPC test of lithium-ion battery; (b) Simplified lithium-ion battery ECM based on EIS analysis and HPPC test.

A hybrid pulse power characteristic (HPPC) test was carried out with a sampling time of 0.1 s, which is commonly used in engineering applications. As can be seen in Fig 3(A), the battery transient response process consists of three stages, i.e., a rapidly rising, a slowly rising, and a slow steady stage. The battery response in the rapidly rising stage could be associated with the EIS response in the high-frequency region. The battery voltage increased rapidly due to Ohmic polarization; this can be simplistically modeled by a resistor, instead of the complex model in the high-frequency region shown in Fig 2. In the slowly rising stage, the battery could be modeled by the parallel combination of a resistor and a CPE, which corresponds to the mid-frequency region in Fig 2. The battery voltage slowly increased in the slow steady stage, which is associated with the low-frequency region in the EIS spectrum. The HPPC test results indicate that the low-frequency plot should be regarded as a part of a depressed semicircle with a large diameter rather than a straight line. Thus, a parallel combination can be used to explain the depressed semicircle in the low-frequency section.

On the basis of the EIS analysis and HPPC test, the battery ECM could be simplified as shown in Fig 3(B). *V*_ser_ denotes the voltage for *R*_ser_, which represents the Ohmic voltage. *V*_1_ represents the concentration polarization voltage, and *V*_2_ denotes the activation polarization voltage.

### FIM

Previous study showed that FOC can be used to design a more accurate system model [22, 29]. FOC is an area of mathematics used for the study of real number order differential and integral calculus, which is a natural extension of the classical integer order calculus. The operator ${}_{a}D{}_{t}^{r}$ is used to represent the FOC, where *r* ∈ *R*.

aDtr={drdtr,r>01,r=0∫atd(φ−r),r<0 (1)

Three definitions are commonly used for FOC, including the Caputo definition, the Riemann-Liouville definition, and the Grünwald-Letnikov (GL) definition. The GL definition was often used to discretize the continuous fractional order equations [22, 30]. The Grünwald-Letnikov FOC is defined as:

$${}_{a}^{G}D_{t}^{r}f(t)=\underset{\begin{array}{c} h\to 0\\ kh=t-a \end{array}}{\lim}h^{-r}\sum_{j=0}^{k}{\left(-1\right)}^{j}\left(\begin{array}{l} r\\ j \end{array}\right)f(t-jh)$$ (2)

where *h* is the sampling period, *k* is the amount of sampling, and $\left(\begin{array}{l} r\\ j \end{array}\right)$ represents the Newton binomial coefficient generalized to real numbers, which can be expressed as $\left(\begin{array}{l} r\\ j \end{array}\right)=\frac{r!}{j!(j-r)!}$.

As an extension of integer order calculus, the presentation of FOC is highly similar to that of integer order differential in a dynamic system. The fractional order differential equation (FODE) is defined as:

$$a_{n}{\text{D}}^{\alpha_{n}}y(t)+a_{n-1}{\text{D}}^{\alpha_{n-1}}y(t)+\cdots +a_{0}{\text{D}}^{\alpha_{0}}y(t)=b_{m}{\text{D}}^{\beta_{m}}u(t)+b_{m-1}{\text{D}}^{\beta_{m-1}}u(t)+\cdots +b_{0}{\text{D}}^{\beta_{0}}u(t)$$ (3)

where *y*(*t*) is the output of system, *u*(*t*) is the input of system, *a*_*i*_ ∈ *R* and *b*_*j*_ ∈ *R* are both coefficients, *i* = 0, 1,···, *n*, and *j* = 0, 1,···, *m*. In addition, the fractional order transfer function can be expressed as:

$$\frac{Y(t)}{U(t)}=\frac{\sum b_{j}{\text{s}}^{\beta_{j}}}{\sum a_{i}{\text{s}}^{\alpha_{i}}}\;,\;i=0,1,\cdots ,n;\;j=0,1,\cdots ,m$$ (4)

The CPEs in Fig 3(B) could be deciphered by fractional order elements [31]:

$$\begin{array}{l} Z_{CPE_{1}}\left(\text{s}\right)=1/\left[C_{1}{\text{s}}^{\alpha }\right]\\ Z_{CPE_{2}}\left(\text{s}\right)=1/\left[C_{2}{\text{s}}^{\beta }\right] \end{array}$$ (5)

where *α* ∈ *R*, 0 ≤ *α* ≤ 1, *β* ∈ *R*, and 0 ≤ *β* ≤ 1 are arbitrary numbers; *C*_1_ ∈ *R* and *C*_2_ ∈ *R* are coefficients.

When *α* = 1 and *β* = 1, *CPE*_1_ and *CPE*_2_ correspond to capacitors with capacitance *C*_1_ and *C*_2_, respectively:

$$\begin{array}{l} {Z_{CPE_{1}}\left(\text{s}\right)|}_{\alpha =1}=1/\left[C_{1}\text{s}\right]\\ {Z_{CPE_{2}}\left(\text{s}\right)|}_{\beta =1}=1/\left[C_{2}\text{s}\right] \end{array}$$ (6)

From the equivalent circuit illustrated in Fig 3(B) and the above analysis, according to the circuit theory, the following equations can be obtained:

$$V_{o}=V_{ocv}+V_{ser}+V_{1}+V_{2}$$ (7)

$$V_{ser}=-R_{ser}\cdot I$$ (8)

$$-I=C_{1}\cdot \Delta^{\alpha }V_{1}+V_{1}/R_{1}=C_{2}\cdot \Delta^{\beta }V_{2}+V_{2}/R_{2}$$ (9)

where Δ^*α*^ is the FOC operator with the fractional order of *α*.

The current *I* is assumed to be positive when the battery is discharging. Thus,

$$V_{o}=V_{ocv}-R_{ser}\cdot I+V_{1}+V_{2}$$ (10)

$$\left\{ \begin{array}{l} \Delta^{\alpha }V_{1}=-I/C_{1}-V_{1}/R_{1}C_{1}\\ \Delta^{\beta }V_{2}=-I/C_{2}-V_{2}/C_{2}R_{2} \end{array}\right.$$ (11)

These equations can be summed up as follows:

$$\{\begin{array}{c} \Delta^{N}x=A\cdot x+B\cdot I\\ y=C\cdot x+D\cdot I \end{array}$$ (12)

where $A=\left[\begin{array}{cc} -1/R_{1}C_{1} & 0\\ 0 & -1/R_{2}C_{2} \end{array}\right]$, $B=\left[\begin{array}{l} -1/C_{1}\\ -1/C_{2} \end{array}\right]$, *C* = [1 1], *D* = [−*R*_*ser*_], $N=\left[\begin{array}{c} \alpha \\ \beta \end{array}\right]$, $x=\left[\begin{array}{l} V_{1}\\ V_{2} \end{array}\right]$, *y* = [*V*_*o*_−*V*_*ocv*_], and *x* ∈ *R*^2^.

### Battery parameters identification based on LSGA

The battery internal parameters are difficult to obtain under non-laboratory conditions due to the complex electrochemical reaction. Many parameter identification methods have been proposed in literatures, such as least squares method [7], recursive algorithm [20], and genetic algorithm [27].

The least square genetic algorithm (LSGA), derived from the combination of the least squares method and the genetic algorithm, was used to identify the internal parameters of the FIM developed above. The basic operations of the algorithm include coding methods, individual fitness evaluation, and genetic operators (such as selection, crossover, and mutation). The individual fitness evaluation is commonly used to determine the probability of individual genetic population, which must be non-negative (i.e., ≥ 0).

An equivalent voltage tracking system was developed for parameter identification, which can be described by the following model:

$$\{\begin{array}{c} \Delta^{N}\hat{x}=A\cdot \hat{x}+B\cdot I\\ \hat{y}=C\cdot \hat{x}+D\cdot I \end{array}$$ (13)

where $\hat{x}=\left[\begin{array}{l} {\hat{V}}_{1}\\ {\hat{V}}_{2} \end{array}\right]$, and $\hat{y}=\left[{\hat{V}}_{o}-{\hat{V}}_{ocv}\right]$. $\hat{x}\in R^{2}$ is the state vector of tracking system, and $\hat{y}$ is the estimated output voltage of tracking system.

The output voltage difference between the tracking system and the battery system is defined as:

$$e=\hat{y}-y$$ (14)

The tracking target of LSGA is represented by the following goal equation:

$$J\left(\theta \right)=\int_{t1}^{t2}\left(e^{\text{T}}\cdot e\right)\text{d}t$$ (15)

The FIM is discretized according to the stochastic theory, and the discrete state space function is obtained as follows:

$$\{\begin{array}{c} \Delta^{N}x_{k+1}=A\cdot x_{k}+B\cdot I_{k}\\ y_{k}=C\cdot x_{k}+D\cdot I_{k} \end{array}$$ (16)

where *I*_*k*_ is the current of battery at the time index *k*, *y*_*k*_ is the working voltage of battery at the time index *k*, and *x*_*k*_ is the state of battery system at the time index *k*.

The FIM expressed by FOC is denoted as:

$$\Delta^{N}x_{k}=\frac{1}{T_{S}^{N}}\sum_{j=0}^{k}{\left(-1\right)}^{j}\left(\begin{array}{c} N\\ j \end{array}\right)x_{k-j}$$ (17)

where *T*_*S*_ is the system sampling time, and $\left(\begin{array}{c} N\\ j \end{array}\right)=\{\begin{array}{cc} 1 & j=0\\ N(N-1)\cdots (N\text{-}j+1)/j! & j>0 \end{array}$.

Thus,

$$\Delta^{N}x_{k+1}=\frac{1}{T_{S}^{N}}\left[x_{k+1}+\sum_{j=1}^{k+1}{\left(-1\right)}^{j}\left(\begin{array}{c} N\\ j \end{array}\right)x_{k+1-j}\right]$$ (18)

The dynamic mathematical model of the identified battery system is obtained:

$$\left\{ \begin{array}{l} x_{k}=T_{S}^{N}A\cdot x_{k-1}+T_{S}^{N}B\cdot I_{k-1}-\sum_{j=1}^{k}{\left(-1\right)}^{j}\left(\begin{array}{c} N\\ j \end{array}\right)x_{k-j}\\ y_{k}=C\cdot x_{k}+D\cdot I_{k} \end{array}\right.$$ (19)

The equivalent voltage tracking system is discretized as follows:

$$\left\{ \begin{array}{l} {\hat{x}}_{k}=T_{S}^{N}A\cdot {\hat{x}}_{k-1}+T_{S}^{N}B\cdot I_{k-1}-\sum_{j=1}^{k}{\left(-1\right)}^{j}\left(\begin{array}{c} N\\ j \end{array}\right){\hat{x}}_{k-j}\\ {\hat{y}}_{k}=C\cdot {\hat{x}}_{k}+D\cdot I_{k} \end{array}\right.$$ (20)

Next, the parameter identification method was aimed at identifying the minimum value of the goal equation:

$$J\left(\theta \right)=\sum_{0}^{N-1}\left\{{\left(y_{k}-{\hat{y}}_{k}\right)}^{T}\cdot \left(y_{k}-{\hat{y}}_{k}\right)\right\}$$ (21)

**Fig 4:**
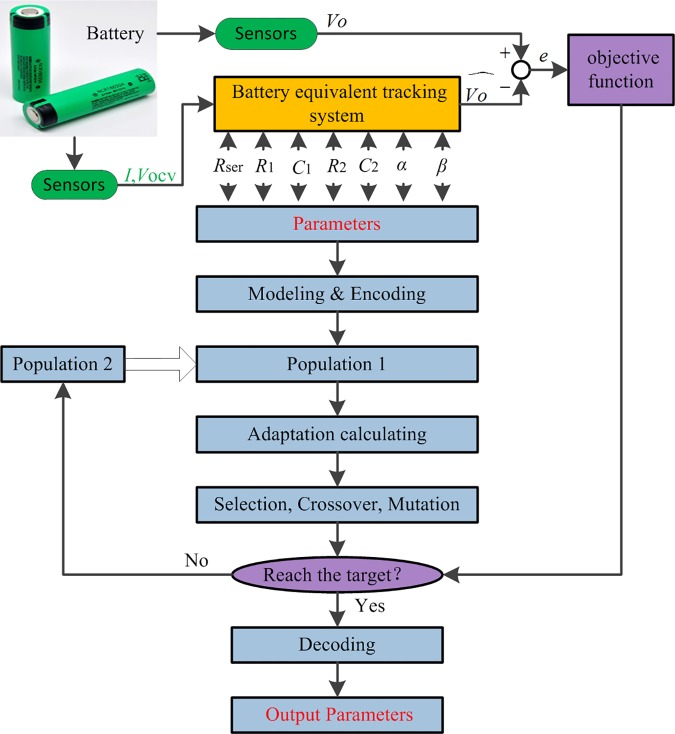
Flow chart of parameter identification.

The flow chart of parameter identification is presented in Fig 4. In which, *I* and *V*_*ocv*_ are the input parameters of the tracking system, *V*_*o*_ is the output voltage of battery, which can be measured directly, and ${\hat{V}}_{o}$ is the output voltage of the tracking system, which can be adjusted via the equivalent tracking model. The batter parameters were identified through the LSGA and FIM developed above, based on the voltage difference between the battery system and the tracking system.

### Results and analysis of parameter identification

**Fig 5:**
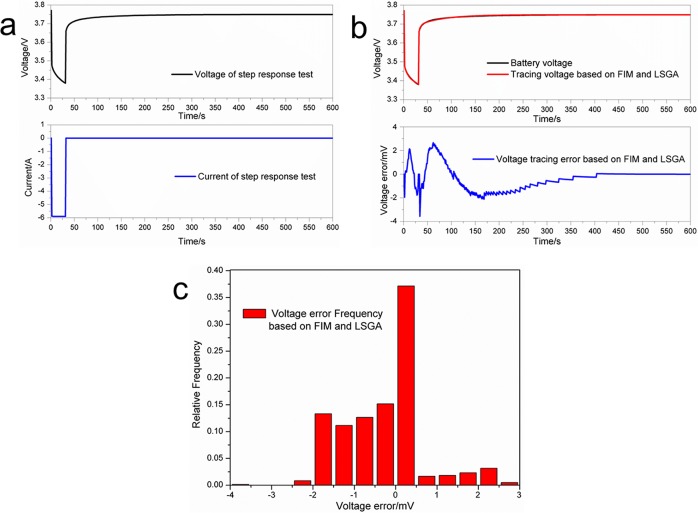
Voltage step response test. (a) Battery voltage and current response; (b) Tracing voltage and tracing error; (c) Voltage tracing error probability.

In order to demonstrate the validity of the proposed model and the designed algorithm, Panasonic NCR18650 batteries were tested via an experimental battery test bench as shown in Figure A in S1 File. In this study, a voltage step response test was performed in order to validate accuracy of the proposed model and parameter identification method. The battery was charged until the voltage reached 3.95 V at a current of 2.065 A. The charging time should be exceeded 10 min in order to ensure a charging balance state, followed by a 30 min rest period. The obtained voltage at the end of rest is regarded as the open circuit voltage (OCV). The test includes, as a key step, an impulse response, which was implemented by discharging the battery with a current of 5.8 A for 30 s. The discharging time was relatively short, so it was considered that the OCV remained unchanged during the test. At the end of the step response test, a 10 min rest was allowed, and the voltage step response was traced using the proposed FIM and LSGA. As shown in Fig 5, the error range of the tracing voltage and the reference voltage can be well confined between –0.004 V and 0.003 V; in particular, the tracing error is mainly in the range between –0.002 V and 0.002 V, and it is close to zero after 400 s. For a more detailed analysis of the voltage tracing results, the voltage tracing error probability was also calculated (Fig 5(C)). These observations indicate that the model and parameter identification method can well characterize the step response of lithium-ion batteries.

**Fig 6:**
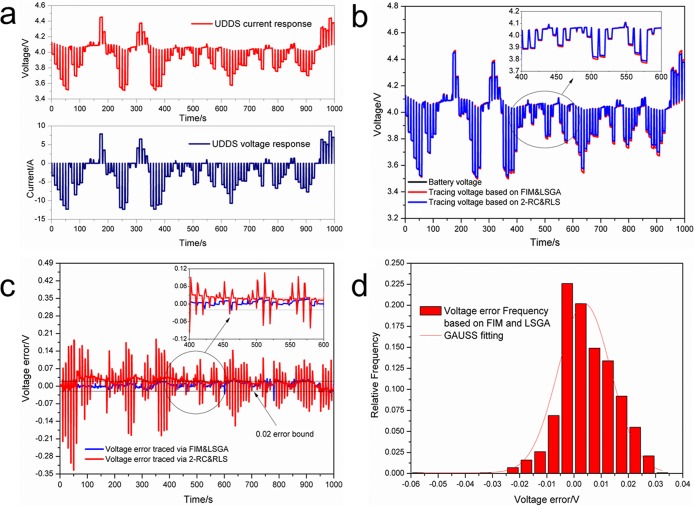
Voltage tracing in UDDS drive cycle test. (a) Battery current and voltage response in UDDS; (b) Voltage tracing of the UDDS test; (c) Voltage tracing error of the UDDS test; (d) Voltage tracing error probability distribution of the UDDS test.

Next, the Urban Dynamometer Driving Schedule (UDDS), a common used driving cycle for EVs, was also performed in order to verify the accuracy of the model online. The scale of the current profile was reduced in accordance with the battery features, and a 1000 s UDDS test was performed to ensure that the OCV remained unchanged (Fig 6). As a comparison, the UDDS test was also carried out using a 2-RC model and the recursive least squares (RLS) method, to demonstrate the superiority of the method proposed in this paper. To simplify the statement, the proposed estimate method based on the fractional model is referred to as a FIM&LSGA method, and the estimate method based on the RC model is referred to as a 2-RC&RLS method. The internal parameters of the batteries were estimated online, and the battery voltage was traced based on the 2-RC&RLS and FIM&LSGA methods (Fig 6(B)), respectively. The tracing voltages were obtained with different accuracy: the fluctuations of the tracing voltage based on the 2-RC&RLS method are larger than those of the tracing voltage based on the FIM&LSGA method. For a more detailed analysis, the tracing errors of the two methods were calculated (Fig 6(C)). Because the battery current changed rapidly and frequently during the UDDS driving cycle, the voltage tracing error is larger than that observed in the voltage step response test. The tracing error curve based on FIM&LSGA is almost a straight line, close to zero, and mostly with an error bound of 0.02 V. On the other hand, the tracing errors based on 2-RC&RLS vary within a large range, even > 0.25 V. Moreover, as can be seen from Fig 6(D), the error distribution of the FIM&LSGA-based method is mostly restricted to the region between –0.015 V and 0.02 V, which corresponds to an error lower than 0.5%; this indicates that the tracing error is small enough for our method to be effectively applied in EV battery management systems.

## Conclusions

In this study, the EIS and HPPC data of lithium-ion batteries were analyzed, and a simplified FIM was developed by introducing a FOC method based on the GL fractional definition. The parameters of the FIM were identified using an equivalent tracking system model through the LSGA. A voltage step response test and a UDDS driving cycle were introduced to assess the performance of the proposed method. The results show that the FIM and parameter identification method can trace the battery work voltage well. Moreover, the voltage tracing error of the proposed method was found to be stabilized at 0.5%, indicating that the FIM and LSGA designed in this work can be applied in EV battery management system. The battery fractional-order model and parameter identification proposed in this study could be used for SOC estimation in the BMS, which is an important performance index of power system for EVs. And the fractional-order parameter sensitivity with battery degradation will be discussed in future based on this study.

## Supporting information

S1 File Table A. Specifications of the lithium-ion batteries used. Figure A. Configuration of the battery test bench.
(DOCX)
