# Supplementary material for: A simplified fractional order impedance model and parameter identification method for lithium-ion batteries
Source: PLoS One. 2017 Feb 17;12(2):e0172424. doi: 10.1371/journal.pone.0172424 (PMC5315307; doi:10.1371/journal.pone.0172424)
Supplement: S1 File — Table A. Specifications of the lithium-ion batteries used. Figure A. Configuration of the battery test bench. (DOCX) [file pone.0172424.s001.docx]

**Electronic Supplementary Information**

**A simplified fractional order impedance model and parameter identification method for lithium-ion batteries**

**Qingxia Yang^1^, Jun Xu^1^, Binggang Cao^1^, Xiuqing Li^2*^**

^1^ State Key Laboratory for Manufacturing Systems Engineering, School of Mechanical Engineering, Xi'an Jiaotong University, Xi'an, Shaanxi, China

^2^ Engineering Research Center of Tribology and Material Protection of Ministry of Education, Henan University of Science and Technology, Luoyang, Henan, China

* Corresponding author

Email: hkdlxq@yeah.net (XL).

**Table A. Specifications of the lithium-ion batteries used.**

| **Type** | NCM lithium-ion battery |  |
| --- | --- | --- |
| **Item** | NCR18650 |  |
| **Nominal Capacity** | 2900 mAh |  |
| **Nominal Voltage** | 3.7 V |  |
| **Operating Voltage** | 2.8V~4.2V |  |
| **Temperature** | Charging: 0~45 °C  Discharging: -20~60 °C |  |


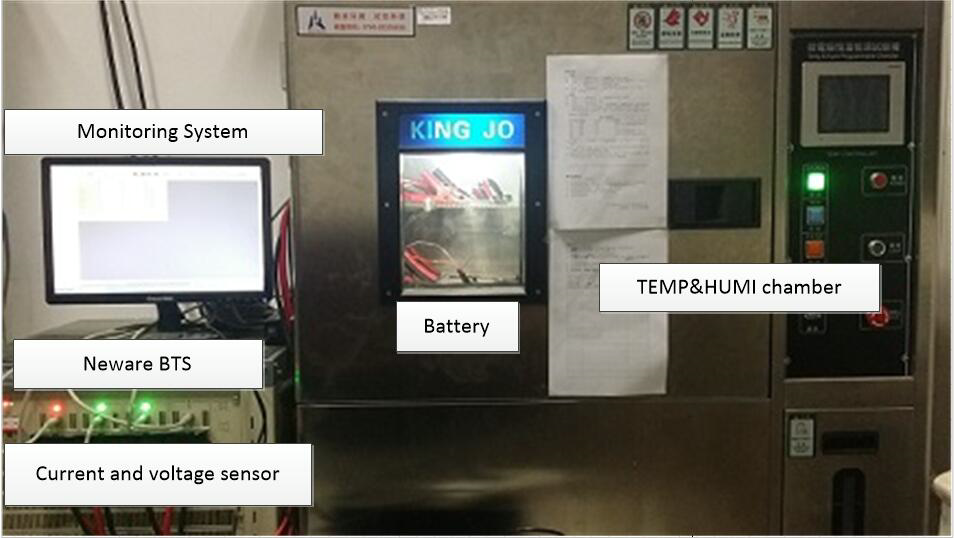


**Figure A. Configuration of the battery test bench**

As can be seen from Figure A, the test bench consists of a computer used as a monitoring system, a Neware battery testing system (BTS), and a temperature and humidity programmable chamber. Battery current and voltage signals are fed back to the computer and processed by the Matlab software for the model internal parameter identification. The temperature and humidity programmable chamber provides a suitable battery test environment (Temperature: 25 °C; Relative humidity: 40%).
